# Prmt7 is dispensable in tissue culture models for adipogenic differentiation

**DOI:** 10.12688/f1000research.2-279.v1

**Published:** 2013-12-18

**Authors:** Yu-Jie Hu, Saïd Sif, Anthony N. Imbalzano

**Affiliations:** 1 Department of Cell & Developmental Biology, University of Massachusetts Medical School, Worcester, MA 01655, USA; 2 Department of Molecular and Cellular Biochemistry, The Ohio State University College of Medicine, Columbus, OH 43210, USA

## Abstract

Protein arginine methylation is a common posttranslational modification that has been implicated in numerous biological processes including gene expression. The mammalian genome encodes nine protein arginine methyltransferases (Prmts) that catalyze monomethylation, asymmetric dimethylation, and symmetric dimethylation on arginine residues. Protein arginine methyltransferase 7 (Prmt7) is categorized as a type II and type III enzyme that produces symmetric dimethylated arginine and monomethylated arginine, respectively. However, the biological role of Prmt7 is not well characterized. We previously showed that Prmt5, a type II Prmt that associates with Brg1-based SWI/SNF chromatin remodeling complex, is required for adipocyte differentiation. Since Prmt7 also associates with Brg1-based SWI/SNF complex and modifies core histones, we hypothesized that Prmt7 might play a role in transcriptional regulation of adipogenesis. In the present study, we determined that the expression of Prmt7 did not change throughout adipogenic differentiation of C3H10T1/2 mesenchymal cells. Knockdown or over-expression of Prmt7 had no effect on lipid accumulation or adipogenic gene expression in differentiating C3H10T1/2 cells or in C/EBPα-reprogrammed NIH3T3 fibroblasts. Based on these results, we conclude that Prmt7, unlike Prmt5, is dispensable for adipogenic differentiation in tissue culture models.

## Introduction

Research over the past 15 years has demonstrated the importance of protein arginine methylation in various biological processes including transcriptional regulation, DNA repair, RNA processing, and signal transduction ^1– 5^. The fact that hundreds of cellular proteins have been identified as the targets of protein arginine methylation supports the idea that arginine methylation regulates diverse cellular processes ^6^. Arginine methylation is catalyzed by protein arginine methyltransferases (Prmts) that transfer a methyl group from *S*-adenosyl methionine (AdeMet) to protein substrates. To date, nine protein arginine methyltransferases have been identified in the mammalian genome and have been classified as type I, type II and type III enzymes by the activity of producing ω- *N ^G^,N ^G^-*asymmetric dimethylarginine (ADMA), ω- *N ^G^,N ^G^-*symmetric dimethylarginine (SDMA), and ω- *N ^G^-*monomethylarginine (MMA), respectively ^2, 4, 7^.

Among the family of Prmts, Prmt7 is unique in that it possesses two AdoMet-binding domains, which may have resulted from a gene duplication event ^8^. As for many of the Prmts, histones are among the identified substrate molecules, suggesting a functional role for Prmt7 in regulating gene expression as a chromatin modifier. Initial work indicated that H2A and H4 were symmetrically dimethylated by Prmt7 *in vitro* ^9^, whereas several studies have reported that Prmt7 only produces MMA on histones and other substrates ^8, 10, 11^. A recent study indicated that Prmt7 symmetrically dimethylates H4R3 and H2AR3 in a manner that contributes to the repression of expression of genes involved in DNA repair ^12^. Another study revealed that Prmt7-mediated H4R3 symmetric dimethylation antagonizes MLL4-catalyzed H3K4 methylation on neuron-specific gene promoters during differentiation ^13^, suggesting that Prmt7 might negatively regulate tissue differentiation by its chromatin-modifying activity. Aside from the role in neuronal differentiation, the regulatory function of Prmt7 in the differentiation of other lineages has not been reported.

Adipocyte differentiation is one of the most intensively studied differentiation processes. Both human and mouse mesenchymal stem cells (MSCs) that reside in fat pads and bone marrow undergo lineage commitment and terminal differentiation to become mature adipocytes ^14– 16^. The adipogenic differentiation process is controlled by a number of tissue-specific transcription factors, such as the CCAAT/enhancer binding proteins (C/EBPs), peroxisome proliferator-activated receptor gamma (PPARγ), and numerous chromatin remodeling and modifying enzymes including the ATP-dependent SWI/SNF complex and Prmts ^17– 20^. It has been shown that Prmt5 interacts with Brg1-based SWI/SNF complex ^21^ and facilitates the binding of Brg1 to the PPARγ and to PPARγ target promoters to facilitate the activation of adipogenic genes ^19^. Furthermore, Prmt4, which also has been shown to interact with Brg1-based SWI/SNF complex ^22^, acts as a co-activator of PPARγ to promote adipocyte gene expression ^20^. Prmt7 had been recently shown to interact with Brg1-based SWI/SNF complex ^12^, but whether Prmt7 has functional roles in adipocyte differentiation remains unclear.

In the present study, we examined the role of Prmt7 in adipocyte differentiation in mouse C3H10T1/2 embryonic mesenchymal cells and in C/EBPα-reprogrammed murine NIH3T3 fibroblasts. By knocking down and over-expressing Prmt7, we showed that Prmt7 has no effect on lipid accumulation and adipogenic gene expression in differentiating cells. Based on the results, we concluded that Prmt7 is not required for differentiation in tissue culture models of adipogenesis.

## Methods

### Cell culture

Mouse C3H10T1/2 and NIH3T3 cells were obtained from the ATCC. C3H10T1/2 cells were maintained in Dulbecco’s modified Eagle’s medium (DMEM) high glucose (Invitrogen) supplemented with 10% fetal calf serum (FCS) (Sigma) and 100U/ml of penicillin/streptomycin (Invitrogen). NIH3T3 cells were maintained in DMEM high glucose with 10% calf serum (Sigma). 293T and BOSC23 cells were obtained from S.N. Jones (UMass Medical School) and R.E. Kingston (Massachusetts General Hospital), respectively, and were grown in the same medium as C3H10T1/2 cells. For adipogenic differentiation, two-day postconfluent cells were differentiated with DMEM medium containing 10% FCS, 10μg/ml insulin, 0.5mM 3-isobutyl-1-methyxanthine, 1μM dexamethasone, and 10μM troglitazone (Sigma). After 48 hours incubation, media on the differentiating cells was replaced with media containing 5μg/ml insulin. Subsequently, the media was changed every other day until harvest. To evaluate cell proliferation, 1×10 ^5^ cells were seeded in 6-well plates (Corning Inc.), and the number of viable cells was counted under a microscope (CK2, Olympus) each day from day 1 to day 4 with a hemocytometer (Hausser Scientific).

### Plasmid DNA constructs

pENTR/pTER+ vector and pLentiX2 Dest vector were gifts from Dr. Eric Campeau (UMass Medical School). The preparation of lentiviral small hairpin RNA (shRNA) constructs was done as previously described ^12, 23^. Briefly, shPrmt7-1, shPrmt7-2 and scrambled control oligonucleotides were cloned into a pENTR/pTER+ vector. These constructs were individually incubated with the pLentiX2 DEST vector and LR clonase II enzyme mix (Invitrogen) to generate pLentiX2 DEST/ shPrmt7-1, pLentiX2 DEST/shPrmt7-2 and pLentiX2 DEST/shCtrl constructs. These lentiviral constructs were amplified in Stbl3 competent cells (Invitrogen) for generating lentiviruses. The pBABE puromycin empty vector (pBABE vector), FLAG-tagged PRMT7 construct (pBABE PRMT7) and pBABE CEBPα construct were previously described ^12, 24^ and were individually amplified in TOP10 competent cells (Invitrogen) for generating retroviral DNA as previously described ^12, 24^.

### Virus production and infection

The preparation of viruses was performed as previously described ^23, 25^. Briefly, for lentiviruses, the packaging vectors pLP1, pLP2, pVSVG (Invitrogen) and pLentiX2 DEST/shRNA constructs were co-transfected into 293T cells with Lipofectamine 2000 reagent (Invitrogen) according to the manufacturer’s instructions. BOSC23 cells were used for pBABE-based retrovirus production. After 48 hours incubation, the supernatant was harvested and filtered through 0.45μm syringe filter (Millipore). For viral infection, 1ml of the filtered supernatant and 4μg/ml of polybrene (Sigma) were used to infect one million cells. After 48 hours incubation, virus infected cells were selected in 2.5μg/ml puromycin (Invitrogen).

### Protein expression analysis

Cells were washed twice with cold PBS and were harvested in RIPA buffer (50mM Tris-HCl pH7.4, 150mM NaCl, 1mM EDTA, 1% Nonidet P-40 (Thermo Scientific) and 0.25% sodium deoxycholate) supplemented with protease inhibitor cocktail (Roche). The samples were sonicated at high intensity setting for 3 minutes with 30sec on/off cycle in a Bioruptor (UCD-200, Diagenode). After quantifying the protein concentration by means of a Bio-Rad protein assay, the protein samples were then mixed with 4× SDS loading buffer (240mM Tris-HCl pH6.8, 8% SDS, 40% glycerol, 0.01% bromophenol blue and 10% β-mercaptoethanol) and boiled at 95°C for 10min. 30μg protein samples were separated on 10% SDS-PAGE and transferred onto PVDF membrane (Bio-Rad). The blots were blocked overnight in 3% non-fat milk (Essential Everyday). The next day, proteins were detected using specific antibodies (1:1000 dilution) and HRP-conjugated secondary antibodies (1:2000 dilution). The rabbit polyclonal antibodies against human PRMT7 (sc-98882) and rat C/EBPα (sc-61) were purchased from Santa Cruz Biotechnology. The mouse monoclonal antibody against mouse PPARγ (sc-7273) and the goat polyclonal antibody against human PRMT5 (sc-22132) were also purchased from Santa Cruz Biotechnology. Rabbit polyclonal anti-PI3K (ABS233) antibody was from Millipore. The secondary antibodies (NA9340 and NA9310) were purchased from GE Healthcare Life Sciences. The blots were developed on X-ray films with ECL Western Blotting Detection Reagents (GE Healthcare Life Sciences). The signal intensity was quantified by ImageJ.

### Gene expression analysis

Total RNA was isolated from samples using TRIzol reagent (Invitrogen) according to the manufacturer’s instructions. cDNA was prepared from 1μg of total RNA by Superscript III reverse transcriptase kit (Invitrogen). Quantitative PCR was performed on StepOne Plus real-time PCR machine with Fast SYBR Green Master mix (Applied Biosystems). The specific primers for gene expression analysis were:

Fasn forward 5′-CGTGTTGGCCTACACCCAGAGCT-3′;

Fasn reverse 5′-GGCAGCAGGGCCTCCAGCACCTT-3′;

AdipoQ forward 5′-CAGTGGATCTGACGACACCA-3′;

AdipoQ reverse 5′-CGAATGGGTACATTGGGAAC-3′;

Fabp4 forward 5′-GCGTGGAATTCGATGAAATCA-3′;

Fabp4 reverse 5′-CCCGCCATCTAGGGTTATGA-3′;

5S rRNA forward 5′-GTCTACGGACATACCACCCTG-3′;

5S rRNA reverse 5′-TACAGCACCCGGTATTCCCAG-3′.

Relative expression levels were determined by the comparative Ct method ^26^.

### Oil Red O staining

Differentiating cells were washed once with PBS and fixed in 10% phosphate-buffered formalin (Fisher Scientific) overnight. The next day, the fixed cells were washed with 60% isopropanol and air-dried completely. The cells were then stained with 60% Oil Red O (AMRESCO) for 10 minutes and washed repeatedly with tap water to remove excess stain. To quantify staining, Oil Red O was extracted from the cells with 100% isopropanol, and the optical density was measured at 500nm (OD _500_).

## Results

### The protein levels of Prmt7 remain constant during adipogenic differentiation of C3H10T1/2 cells

**Figure 1.**
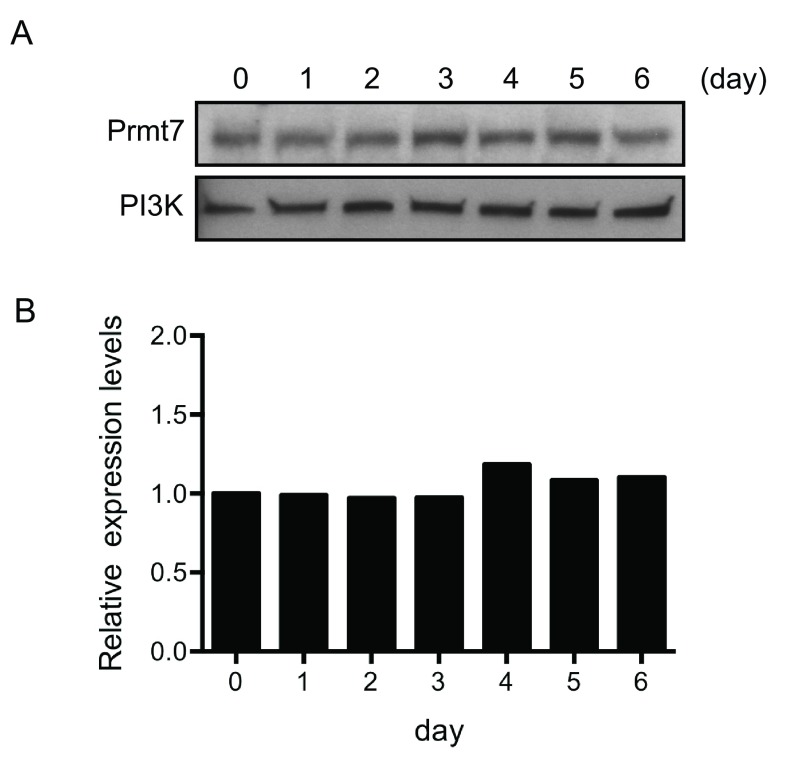
Prmt7 protein levels are constant in differentiating C3H10T1/2 cells. ( **A**) Western blot analysis of the protein extracts from day 0 to day 6 post-differentiated C3H10T1/2 cells. Duplicate blots were probed with anti-Prmt7 antibody and with anti-PI3K antibody as a loading control. ( **B**) The quantification of ( **A**) by ImageJ. The levels of Prmt7 were normalized to PI3K loading control and presented as the relative expression levels to the day 0 sample (day 0=1). The data represent the average of two independent experiments (n=2).

The C3H10T1/2 cell line was established from C3H mouse embryos and has served as a faithful cell culture model for mesenchymal lineage differentiation ^27– 29^. C3H10T1/2 cells can be differentiated into mature adipocytes by treating the confluent cells with a cocktail that contains insulin, dexamethasone, 3-isobutyl-1-methyxanthine (IBMX) and PPARγ ligands ^30^. Using this model, we first examined Prmt7 protein levels during adipogenic differentiation by Western blot analysis. We found that Prmt7 protein levels are relatively constant from the onset of differentiation (day 0) through the day 6 post-differentiation ( Figure 1A and Figure 1B) ( Dataset 1). We concluded that Prmt7 protein levels were not altered in differentiating C3H10T1/2 cells.

### Virus-mediated knockdown and over-expression of Prmt7 in C3H10T1/2 cells

**Figure 2.**
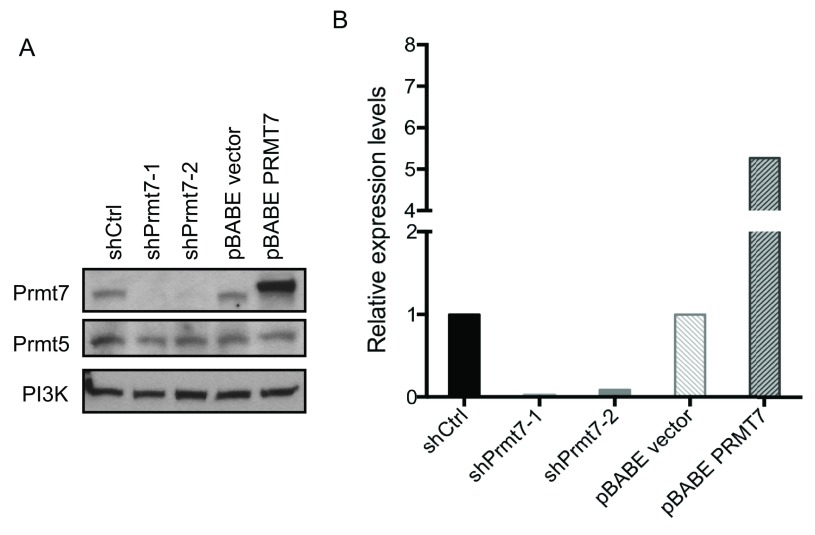
Prmt7 was specifically knocked down or over-expressed in C3H10T1/2 cells. ( **A**) A representative Western blot analysis from proliferating C3H10T1/2 cells with Prmt7 knockdown and over-expression. Endogenous Prmt7 was specifically depleted by two different lentiviral shRNA constructs (shPrmt7-1 and shPrmt7-2). The scrambled shRNA lentiviral construct (shCtrl) was used as a control. The pBABE retroviral construct encoding FLAG-tagged PRMT7 (pBABE - PRMT7; ^12^) was used to ectopically express PRMT7, and the pBABE empty vector (pBABE vector) was used as a control. Duplicate blots were probed with anti-Prmt7 and anti-Prmt5 antibodies and with anti-PI3K antibody as a loading control. ( **B**) The quantification of Prmt7 in ( **A**) by ImageJ. The levels of Prmt7 were normalized to the PI3K loading control and are presented as expression levels relative to the scrambled shRNA control or pBABE empty vector control. The data represent the average of two independent experiments (n=2).

To study the function of Prmt7, we used viral vectors to knock down or over-express Prmt7 in C3H10T1/2 cells. Two lentiviral constructs (pLentiX2 DEST/shPrmt7-1 and pLentiX2 DEST/shPrmt7-2) that encode shRNAs against Prmt7 mRNA were used for knocking down endogenous Prmt7 in proliferating C3H10T1/2 cells. A pBABE retroviral construct (pBABE-PRMT7) encoding FLAG-tagged PRMT7 was used to over-express PRMT7. The virus-infected cells were selected with puromycin and the levels of Prmt7 in the selected cells were examined by Western blot analysis ( Figure 2A). Endogenous Prmt7 levels were reduced 10-fold or more in the knockdown cells compared to the scrambled shRNA control cells ( Figure 2B). Prmt7 levels were increased more than 5-fold in the FLAG-tagged PRMT7 over-expression cells compared to the pBABE empty vector control ( Figure 2B) ( Dataset 2). Since Prmt5 is the major type II arginine methyltransferase and is also associated with SWI/SNF complexes ^21^, we examined Prmt5 protein levels in our samples. We observed no changes in Prmt5 levels in Prmt7 knockdown and over-expression C3H10T1/2 cells ( Figure 2A).

### Prmt7 has no effect on cell proliferation of C3H10T1/2 cells

**Figure 3.**
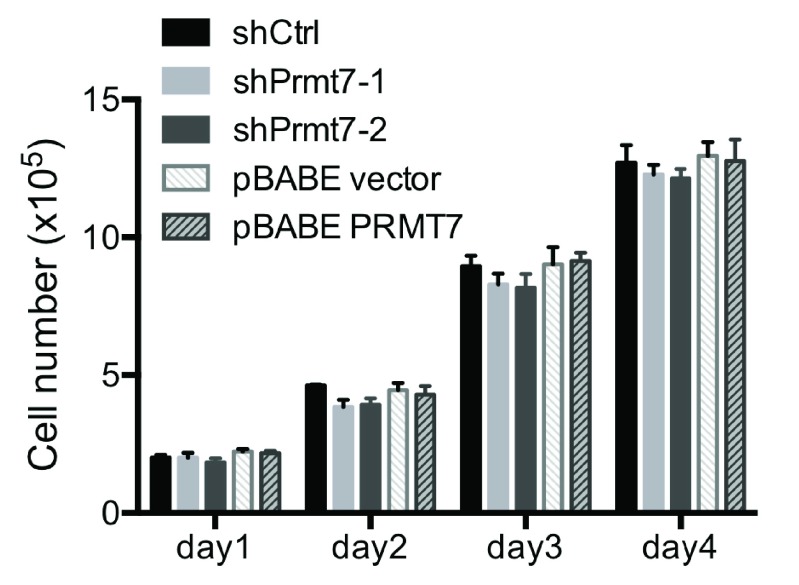
Knockdown or over-expression of Prmt7 does not affect C3H10T1/2 cell proliferation. The growth rates of control (shCtrl and pBABE vector), Prmt7 knockdown (shPrmt7-1 and shPrmt7-2) and Prmt7 over-expression (pBABE PRMT7) C3H10T1/2 cells were measured by seeding 1×10 ^5^ cells in 6-well plates, and the viable cells were counted each day for 4 days after seeding. The data represent the average of two independent experiments (n=2) counted in duplicate. Error bars show the standard deviation.

Both Prmt7 and Prmt5 exhibit type II arginine methyltransferase activity. However, unlike Prmt5, Prmt7 has no effect on cell proliferation in NIH3T3 fibroblasts ^12^. We measured the cell proliferation rate of Prmt7 knockdown and over-expression C3H10T1/2 cells, and found that neither the reduction of Prmt7 nor the over-expression of Prmt7 affected the proliferation of C3H10T1/2 cells ( Figure 3) ( Dataset 3). This result is consistent with the results from the previous study on NIH3T3 fibroblasts ^12^.

### Prmt7 is not required for adipogenic differentiation of C3H10T1/2 cells

**Figure 4.**
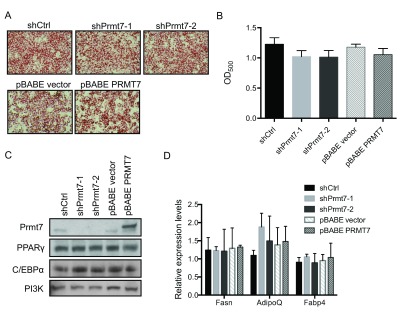
Knockdown or over-expression of Prmt7 has no effect on C3H10T1/2 adipogenic differentiation. ( **A**) Representative Oil-Red O staining images of day 6 post-differentiated C3H10T1/2 cells in which Prmt7 was either knocked down (shPrmt7-1 and shPrmt7-2) or over-expressed (pBABE PRMT7). The Prmt7 knockdown and over-expression C3H10T1/2 cells were grown to confluence and differentiated 48 h later. At day 6 post-differentiation, the cells were fixed with 10% formalin and stained with Oil-Red O. ( **B**) The quantification of ( **A**). The Oil-Red O stain was extracted with 100% isopropanol and the optical density at 500nm (OD _500_) was determined. The data represent the average of two independent experiments (n=2) assayed in duplicate. Error bars show standard deviation. ( **C**) A representative Western blot analysis of endogenous Prmt7, PPARγ and C/EBPα expression in day 6 post-differentiated C3H10T1/2 cells. The levels of PI3K are presented as a loading control. ( **D**) Gene expression analysis on day 6 post-differentiated C3H10T1/2 cells. The mRNA levels of fatty acid synthase ( *Fasn*), adiponectin ( *AdipoQ*) and fatty acid binding protein 4 ( *Fabp4*) were measured by RT-qPCR. The individual mRNA levels were normalized to 5S rRNA. The normalized expression levels of the control cells in one of the experiments were set as 1. The data are presented as the average of relative expression levels from two independent experiments (n=2) assayed in duplicate. Error bars show standard deviation.

To determine whether Prmt7 affects adipogenesis, the Prmt7 knockdown and over-expression C3H10T1/2 cells were grown to confluence and treated with the differentiation cocktail. At day 6 post-differentiation, the accumulation of intracellular neutral lipids was measured by Oil Red O staining. The Oil Red O staining showed similar levels of lipid accumulation in Prmt7 knockdown as well as Prmt7 over-expression cells as compared to the control cells. ( Figure 4A and Figure 4B) ( Dataset 4). This result suggests that the changes in Prmt7 levels did not affect lipid accumulation in differentiating C3H10T1/2 cells.

### Adipogenic gene and protein expression in C3H10T1/2 cells was not affected by Prmt7 knockdown or over-expression

PPARγ and C/EBPα are the key transcription factors for adipogenic differentiation and for the maintenance of the adipocyte phenotype ^31– 34^. We examined the protein levels of PPARγ and C/EBPα in day 6 post-differentiation cells by Western blot analysis and found no significant difference in either Prmt7 knockdown or Prmt7 over-expression cells compared to the corresponding controls ( Figure 4C). In addition, to rule out the possibility that Prmt7 functions as a cofactor of PPARγ and C/EBPα, we measured the mRNA expression levels of PPARγ and C/EBPα target genes in day 6 post-differentiation samples by real-time quantitative PCR ( Figure 4D) ( Dataset 5). We found that Prmt7 had no significant impact on fatty acid synthase ( *Fasn*), adiponectin ( *AdipoQ*), and fatty acid binding protein 4 ( *Fabp4*) gene expression in the differentiating cells. These results suggest that Prmt7 is dispensable for adipogenic gene expression.

### Prmt7 has no effect on C/EBPα-reprogrammed NIH3T3 fibroblasts

**Figure 5.**
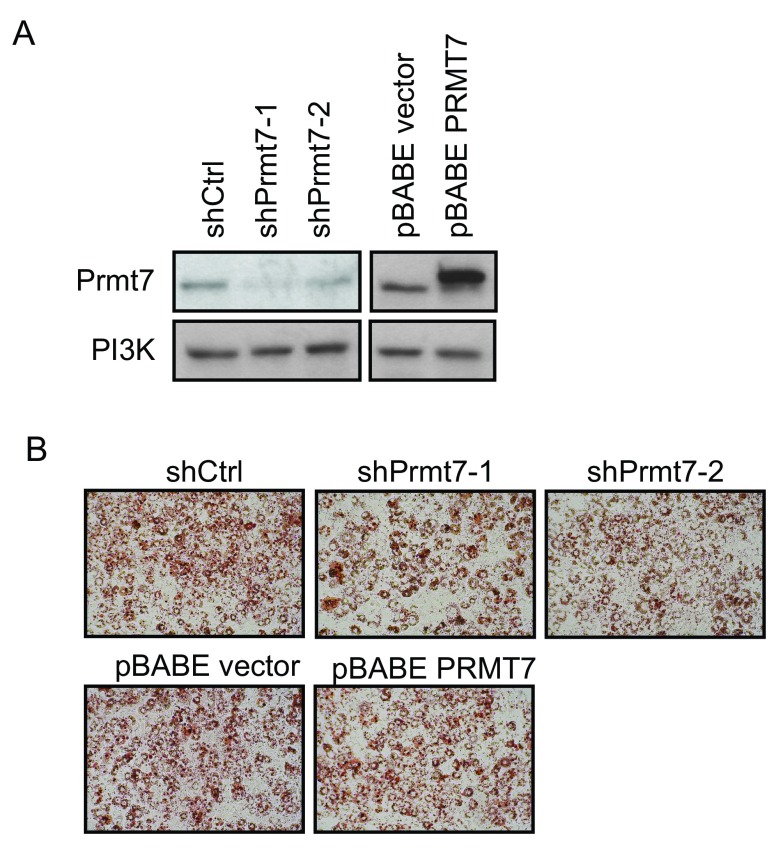
Knockdown or over-expression of Prmt7 has no effect on C/EBPα-mediated reprogramming of NIH3T3 fibroblasts. ( **A**) Western blot analysis on Prmt7 knockdown and over-expression NIH3T3 fibroblasts. Endogenous Prmt7 was specifically depleted by the lentiviral shRNA constructs (shPrmt7-1 and shPrmt7-2). The scrambled shRNA lentiviral construct (shCtrl) was used as a control. The pBABE retroviral construct encoding FLAG-tagged PRMT7 (pBABE PRMT7) was used to ectopically express PRMT7 and the pBABE empty vector was used as a control. The blot was probed with anti-Prmt7 antibody, and the PI3K levels are presented as a loading control. ( **B**) Oil-Red O staining images of C/EBPα-reprogrammed NIH3T3 fibroblasts at day 6 post-differentiation. Prmt7 knockdown and over-expression NIH3T3 fibroblasts were infected with retroviruses encoding C/EBPα at 70% confluence. Two day post-confluent cells were differentiated. At day 6 post-differentiation, the cells were fixed with 10% formalin and stained with Oil-Red O.

Previous studies had shown that ectopic expression of PPARγ or C/EBPα alone in non-adipogenic NIH3T3 cell line is able to reprogram NIH3T3 fibroblasts into adipocyte-like cells ^33, 34^. To test if Prmt7 is required for the reprogramming of NIH3T3 fibroblasts, we first knocked down and over-expressed Prmt7 in NIH3T3 fibroblasts by using the same viral constructs that we used in C3H10T1/2 cells, and confirmed the knockdown and over-expression of Prmt7 by Western blot analysis ( Figure 5A). These cells were further infected with retroviruses encoding C/EBPα at 70% confluence. After the cells reached confluence, the differentiation cocktail was added to stimulate adipogenic differentiation. At day 6 post-differentiation, the accumulated lipid was evaluated by Oil Red O staining ( Figure 5B). We found that neither knockdown nor over-expression of Prmt7 caused a significant difference in Oil Red O staining in C/EBPα-reprogrammed NIH3T3 fibroblasts, which is consistent with the results in C3H10T1/2 cells.

Data files Prmt7 and adipocyte differentiation Dataset 1: Dataset for Fig.1B Quantification of Prmt7 protein levels in differentiating C3H10T1/2 cells. Column A describes the time when cells were harvested. Columns B to E show the signal intensities of Prmt7 and PI3K from two independent experiments, quantified by ImageJ. Columns F and G show the ratio of Prmt7 levels to PI3K levels for each sample in each experiment. Columns H and I show relative Prmt7 levels where the day 0 sample in each experiment is normalized to 1. Column J shows the mean of the relative Prmt7 levels from the two independent experiments.
Dataset 2: Dataset for Fig.2B Quantification of Prmt7 protein levels in the knockdown and over-expression C3H10T1/2 cells. Column A shows the names of the cells. Columns B to E show the signal intensities of Prmt7 and PI3K from two independent experiments, quantified by ImageJ. Columns F and G show the ratio of Prmt7 levels to PI3K levels for each sample in each experiment. Columns H and I show relative Prmt7 levels where the shControl and the pBABE vector sample in each experiment is normalized to 1. Column J shows the mean of the relative Prmt7 levels from the two independent experiments.
Dataset 3: Dataset for Fig.3 Cell counting results. Column A shows the time when cell numbers were counted. Column B shows the names of the cells. Columns C to F show the numbers of the cells (x105) from two independent experiments with two replicates in each experiment. Column G shows the mean of the cell numbers. Column H shows the standard deviation (SD) of the cell numbers.
Dataset 4: Dataset for Fig.4B Quantification of Oil-Red O staining on day 6 post-differentiated C3H10T1/2 cells. Column A shows the names of the cells. Columns B to Column E show the values of OD500 from two independent experiments with two replicates in each experiment. Column F represents the mean of the OD500 values. Column F shows the mean values. Column G shows the standard deviation (SD) of the OD500 values.
Dataset 5: Dataset for Fig.4D Real-time qPCR results for the expression of selected genes in day 6 post-differentiated C3H10T1/2 cells. Column A shows to the names of the transcripts. Column B shows the names of the cells. Column C indicates the experiment number. Columns D and E show raw Ct values from two replicates from each sample. Column F shows the mean raw Ct value. Columns G to K show the following values: delta Ct (Ct – Ct5S rRNA), delta delta Ct (dCt - dCtshCtrl), fold change (2^(-ddCt)), the mean of the fold change, and the standard deviation (SD) of the fold change.

## Discussion

Changes in gene expression during cell differentiation require alterations in higher-order chromatin organization as well as in local chromatin structure. Cells possess histone modifying enzymes and ATP-dependent chromatin remodeling enzymes to facilitate chromatin changes. The interplay between these two families of enzymes has been shown to be crucial for both transcription activation and repression ^18, 21, 35^. Prmt7 was identified as a histone arginine methylating enzyme ^9– 11^ and was shown to associate with Brg1-based SWI/SNF ATP-dependent chromatin remodeling complex ^12^. These findings led us to investigate the possible roles of Prmt7 in adipogenic differentiation, which is a process that requires the function of Brg1-based SWI/SNF complex ^24^. Our data clearly showed that Prmt7 levels were significantly changed in the knockdown or over-expression cells, but manipulation of Prmt7 levels did not cause a differentiation deficiency. It is established that Brg1-based SWI/SNF complex is recruited to the adipogenic promoters upon differentiation ^24^. However, whether Prmt7 associates with Brg1-based SWI/SNF complex at adipogenic promoters is still unknown. Since Prmt7 has no effect on adipogenic gene expression, we expect that Prmt7 is not recruited to adipogenic promoters. Alternatively, even if there is binding, the function of Prmt7 is dispensable at these loci.

Functional redundancy within the Prmt family has not been characterized. Prmt7 was classified as a type II and a type III arginine methyltransferase by characterization of its *in vitro* catalytic activity ^8– 11^. Whether other Prmts functionally compensate for Prmt7 is still unknown. The predominant type II arginine methyltransferase Prmt5 catalyzes the formation of MMA and SDMA in a nonprocessive fashion ^36, 37^. Type I arginine methyltransferases also produce MMA ^38– 40^. It is possible that Prmt5 or type I Prmts partially or fully compensate for the loss of Prmt7 in the cells. Our data showed that Prmt7 knockdown or over-expression has no effect on Prmt5 protein levels in C3H10T1/2 cells. This observation is consistent with the results from the previous study in HeLa cells ^41^. However, we still cannot rule out the possibility that Prmt5 compensates for Prmt7 enzyme activity, even though Prmt5 protein levels remain constant. Further investigation is needed to address the possible crosstalk between Prmt5 and Prmt7 in chromatin regulation.

To our knowledge, Prmt7 knock-out or transgenic mice have not been reported. Whether changes in Prmt7 levels cause any developmental deficiencies *in vivo* remains unknown. However, several studies using cell lines or tissues have revealed regulatory roles for Prmt7 in tissue-specific gene expression. For example, PRMT7 negatively regulates neuronal differentiation of a human embryonal carcinoma cell line by repressing the expression of differentiation-specific genes ^13^. In mouse germ cells, Prmt7 was recruited to the imprinting control region through physical interaction with CTCFL, a testis-specific nuclear protein, and repressed imprinted gene expression ^42^. Furthermore, mouse embryonic stem cells and germ cells have relative high levels of Prmt7 compared with mouse embryonic fibroblasts ^43, 44^. This evidence suggests that Prmt7 might have important functions in the maintenance of stem cell pluripotency and that the down-regulation of Prmt7 might be required for early cell fate determination. Taken together, these data suggest that Prmt7 might have a role during early development.
