# Supplementary material for: Prmt7 is dispensable in tissue culture models for adipogenic differentiation
Source: F1000Res. 2013 Dec 18;2:279. [Version 1] doi: 10.12688/f1000research.2-279.v1 (PMC3962006; doi:10.12688/f1000research.2-279.v1)
Supplement: Peer review history

# Peer review: Referee response for version 1

**Published:** 2014-02-13

**Authors:** Yaacov Barak

**Affiliations:** 1 Department of Pathology, Magee-Womens Research Institute and the University of Pittsburgh, Pittsburgh, PA, USA

In this manuscript, Hu *et al.* performed a series of high-quality, well-designed and properly controlled experiments in search of potential functions of the protein arginine methylase Prmt7 in adipogenesis of C3H10T1/2 cell cultures. The motivation for the experiment was the group’s previous work that identified central functions of the closely related Prmt5 in the process, which led them to hypothesize guilt-by-homology. Moreover, hints of developmental functions of Prmt7 in other systems, such as neuronal differentiation, warranted testing a broader effect on differentiation in other system.

 

The conclusion that Prmt7 has no bearing on adipogenesis is fully justified by the clean data, which showed no effect in both loss and gain-of-function approaches. It is reassuring that the experiment was conducted by the same group that identified the roles of Prmt5 in the process, and thus the negative data are likely not due to experimental system variability.

 

In the Discussion, the authors allow that this lack of effect may be the result of compensation by redundant methylases; this is probably an overly cautious, “just-in-case” argument, considering that even in most cases of true redundancy there are at least some quantitative effects, which is not the case here whatsoever. It is more likely that arginine methylases are target-specific and have a clear partition of labor among them.

 

With heaps of negative data buried in the archives of many laboratories which never see the light of day, the team has to be commended for publishing their own negative data to seal the issue and allow other research groups that might be drawn to the question to steer away and focus on more productive hypotheses.

I have read this submission. I believe that I have an appropriate level of expertise to confirm that it is of an acceptable scientific standard.

# Peer review: Referee response for version 1

**Published:** 2014-01-27

**Authors:** Jeffrey Gimble

**Affiliations:** 1 Center for Stem Cell Research and Regenerative Medicine, Tulane University, New Orleans, LA, USA

This is a well designed, well conducted, and well described study from a leading lab in the evaluation of histone mediated transcriptional regulation of adipogenesis. The authors provide compelling evidence that Prmt-7 is not directly involved in adipogenic regulation using loss of function/gain of function approaches in two independent pre-adipocyte models *in vitro*. The authors have evaluated their findings objectively and placed it appropriately in the context of the existing literature. The manuscript and figures are of top quality and require no further modification. The findings provide information that will benefit others in the scientific community by documenting the absence of a direct relationship between Prmt-7 and adipogenic mechanisms.

I have read this submission. I believe that I have an appropriate level of expertise to confirm that it is of an acceptable scientific standard.
